# Luteolin enhances erlotinib’s cell proliferation inhibitory and apoptotic effects in glioblastoma cell lines

**DOI:** 10.3389/fphar.2022.952169

**Published:** 2022-09-19

**Authors:** Erika Powe, Daniel Parschauer, Jessica Istifan, Stacy Lin, Huanyun Duan, Rebecca Gryka, Denise Jean-Louis, Amit K. Tiwari, Samson Amos

**Affiliations:** ^1^ Department of Pharmaceutical Sciences, Cedarville University School of Pharmacy, Cedarville, OH, United States; ^2^ Department of Pharmacology and Experimental Therapeutics, Toledo, OH, United States; ^3^ Department of Cell and Cancer Biology, University of Toledo, Toledo, OH, United States; ^4^ Center of Medical Bio-Allied Health Sciences Research, Ajman University, Ajman, UAE

**Keywords:** EGFR signaling inhibitors, glioblasoma multiforme, apoptosis, signaling, signaling pathways, antioxi dant

## Abstract

The epidermal growth factor (EGFR) receptor is frequently overexpressed in glioblastoma multiforme IV (GBM). Increased expression of EGFR leads to increased proliferation, decreased apoptosis, and increased resistance to chemotherapeutic agents. A small molecule called erlotinib inhibits EGFR receptors by binding to their adenosine triphosphate (ATP) binding sites. It is FDA approved to treat a variety of EGFR-mediated cancers. Several clinical trials have explored a combination of erlotinib with other agents to treat glioblastoma since it is believed that erlotinib would benefit patients with GBM with EGFR mutations or expression. Luteolin, a natural flavonoid, inhibits cell growth and induces apoptosis in cancer cells. We investigated the combined effects of erlotinib and luteolin on proliferation and apoptosis on glioblastoma cell lines overexpressing EGFR or glioma cells expressing truncated EGFR (ΔEGFR). In a concentration-dependent fashion, the combination of luteolin and erlotinib reduced cell proliferation (*p* < 0.05) and induced apoptosis by cleaving PARP and increasing caspase expression. In addition, the combination of luteolin and erlotinib reduced the phosphorylation of downstream EGFR cell signaling molecules such as Akt, NF kappa B, and STAT3 in a concentration-dependent manner. These findings suggest that combining luteolin with erlotinib offers a potential treatment strategy for glioblastoma multiforme IV.

## Introduction

The glioblastoma multiforme grade IV (GBM) is a malignant tumor that affects ten out of every 100,000 adults each year (Iacob and Dinca 2009; Siegel et al., 2021). Among all primary brain tumors, GBMs account for 10–15 percent of all glial tumors. GBM presents with a poor prognosis of about 37% in 1 year and 5-year survival of 5% (Krex et al., 2007; Tan et al., 2018). Patients’ survival rates are low, with the average survival time after diagnosis being approximately 14 months (Krex et al., 2007). Low survival rates, decreased apoptosis, increased proliferation, and cell migration are some of the hallmarks of glioblastoma. GBM is a heterogeneous tumor, which means that every cell within the tumor exhibits different characteristics, making treatment challenging. The most common treatments are surgical removal of tumors, radiation therapy, and/or chemotherapy using temozolomide, an alkylating agent (Friedman et al., 2000).

**FIGURE 1:**
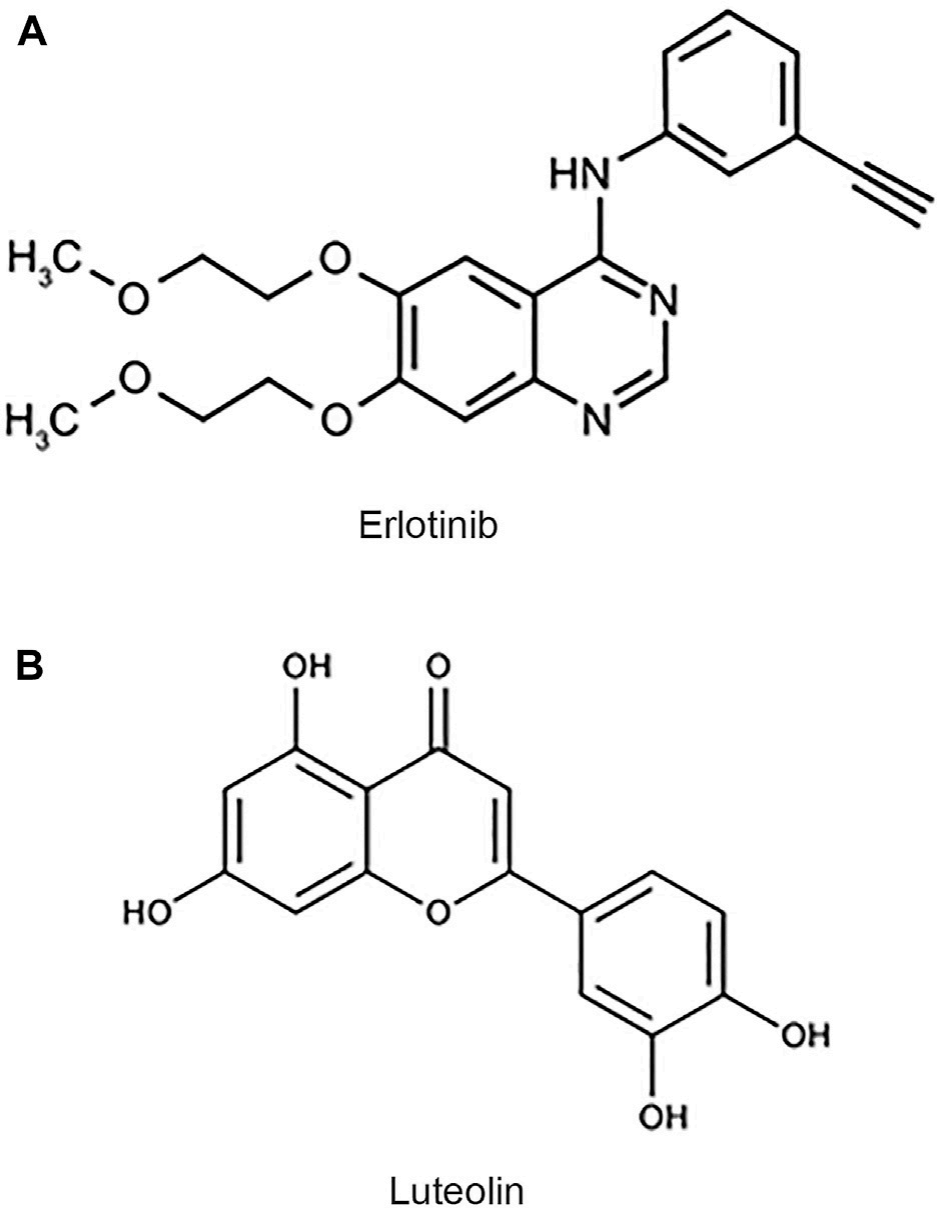
Chemical structures of erlotinib and luteolin. **(A)** Chemical structure of erlotinib, a small molecule inhibitor of EGFR. **(B)** Chemical structure of luteolin, a flavonoid derived from vegetable sources.

The epidermal growth factor receptor (EGFR) is an important factor in GBM since growth factors and their receptors are primarily responsible for regulating cell proliferation (Padfield et al., 2015). There is an abnormal expression of EGFR in 40–60 percent of patients with GBM. The EGFR overexpression contributes to the highly proliferative and treatment-resistant nature of this tumor. Due to poorly understood mechanisms, the heterogeneity of the tumor can allow the cells to reversibly increase or decrease their level of EGFR vIII expression, maximizing their growth potential (Haas-Kogan et al., 2005; Padfield et al., 2015). Erlotinib (Figure 1A), is an EGFR tyrosine kinase inhibitor that prevents intracellular phosphorylation of EGFR in cancer cells, preventing downstream signaling and causing cancer cell death (Haas-Kogan et al., 2005). It shows promise in treating gliomas (Clarke et al., 2014; Raizer et al., 2016). Resistance to erlotinib appears to be linked to EGFR vIII suppression in extrachromosomal DNA (Haas-Kogan et al., 2005; Padfield et al., 2015). As erlotinib is clinically approved for other indications, enhancing its efficacy with the right adjuvants will increase its effectiveness and utility in treating GBM.

Luteolin (Figure 1B) is a common flavonoid found in various plants, fruits, vegetables, and medicinal herbs. Flavonoids are some of the most abundant compounds found in the human diet (Sharma et al., 2011). Flavonoids display various beneficial effects such as antioxidant, anticancer, anti-inflammatory, and antiviral properties. It has been shown that luteolin’s antiproliferative property is due to its ability to arrest the cell cycle at checkpoints, G1/s, and G2/M (Lin et al., 2008). The anticancer property of luteolin is of specific interest because it has been shown to induce apoptosis, inhibit cell proliferation, and inhibit metastasis and angiogenesis (Sawmiller et al., 2014; Anson et al., 2018). Furthermore, luteolin can sensitize cancer cells by suppressing cell survival via the NF-kB and the PI3K/Akt pathways (Sawmiller et al., 2014; Anson et al., 2018). Luteolin is a flavonoid that has been proposed to cross the blood-brain barrier (BBB; Youdim et al., 2004; Sawmiller et al., 2014).

Hormesis is a phenomenon in which a substance is stimulatory at low concentrations and inhibitory at higher concentrations (Calabrese et al., 2007; Calabrese et al., 2010; Calabrese et al., 2012). Hormesis has been observed in many biological systems (Drake et al., 2003; Calabrese et al., 2007). The hormetic dose-response exhibited by luteolin and other flavonoids in a range of studies was assessed and reported recently (Suraweera et al., 2020; Calabrese et al., 2021). The studies indicate that at low doses luteolin may be cytoprotective and cytotoxic at higher doses. At the doses of luteolin used in our study, cytotoxicity was observed. The biphasic hormetic response reported with luteolin indicates that this compound has the potential to be effective at chemoprevention when incorporated into a healthy diet as well as improve the chemotherapeutic efficacy of agents such as erlotinib.

The mechanisms by which luteolin and erlotinib inhibit cell proliferation and induce apoptosis are unknown. We investigated the anticancer potential of EGFR inhibitors such as erlotinib in combination with luteolin. These combinatorial effects could lead to a decrease in intracellular survival signaling mediated by the overexpression of the epidermal growth factor receptors.

## Materials and methods

### Cell lines

Cell lines U251MG and U87 ΔEGFR were supplied by Dr. Isa Hussaini, Department of Pathology, University of Virginia, Charlottesville, VA. The U87 cell line was purchased from ATCC (Manassas, VA). The U251 EGFR and EGFR vIII were a kind gift from Dr. James Mandell, Department of Pathology, University of Virginia, Charlottesville, VA. The cell lines were maintained in DMEM media supplemented with 10% fetal bovine serum (FBS) and 1% penicillin and streptomycin. All cells were incubated at 37°C in a humidified incubator at 5% CO_2_.

### Materials

Erlotinib was purchased from Cayman Chemical Company (Ann Arbor, MI). Luteolin was purchased from Indofine Chemical Company (Hillsborough, NJ). Primary antibodies to Akt, ERK1/2, mTOR, p70S6K, PARP, and Bcl-xL were purchased from Cell Signaling Technology (Danvers, MA). Tubulin antibody was purchased from Sigma (St. Louis, MO). MTT assay kits were obtained from Promega (Madison, WI). DMEM and penicillin/streptomycin antibiotics were purchased from Gibco (Carlsbad, CA). Annexin V/FITC staining kit was purchased from Thermo Fisher (Waltham, MA).

### MTT assay

Cell viability was evaluated after treatment with luteolin in combination with erlotinib. An MTT assay was utilized to assess the effects of varying concentrations of erlotinib in the absence or presence of luteolin (0, 10, and 20 µM) after incubation for 3 days. After incubation, 10 µL of MTT dye was added to each well and incubated for 4 hours at 37°C. After the 4 h incubation, 100 µL of solubilization reagent (40% v/v dimethylformamide in 2% glacial acetic acid to which 16% w/w SDS was added, and pH adjusted to 4.7) was added to each well. Plates were then rocked and covered with foil for 1 hour at room temperature. The potential of cells to reduce the MTT to formazan was determined using the Promega Glomax Multi Detection System at a wavelength of 560 nm. The rate of viable cell was calculated using the optical density. The mean ± SEM for each experiment was calculated and plotted using GraphPad Prism version 9. This experiment was done in triplicates (Stump et al., 2017; Han et al., 2020).

### Combination index assay

We utilized the drug combination of luteolin and erlotinib in glioblastoma cells for the combination index assay. Combination indices (CI) were generated according to the Chou–Talalay method (Chou, 2010). The equation for the classic isobologram is given by: CI= (D)_1_/(Dx)_1_ + (D)_2_/(Dx)_2_. Where (Dx)1 and (Dx)2 indicate the individual dose of luteolin and erlotinib required to inhibit a given level of cell growth and D1 and D2 indicate the doses of luteolin and erlotinib necessary to produce the same effect in combination, respectively. The combined response of both agents can be summarized as CI < 1, CI = 1, and CI > 1, indicating synergistic, additive, and antagonistic effects, respectively.

### Colony formation assay

The ability of U251 glioma cells to reproduce and survive following treatment with luteolin and erlotinib was assessed using the colony formation assay as described by Zhao et al., 2018. Briefly, U251 glioma cells were seeded at a density of 5 × 10^2^ cells in a 6-well plate for 24 h. After the cells adhered, the cells were treated with either luteolin or erlotinib or a combination of both agents for 16 h. The media was changed to media plus FBS and replaced every 4 days for 10–14 days. After 14 days, the surviving clones were fixed with 4% paraformaldehyde and stained with 0.01% crystal violet for 30 min at room temperature. The excess crystal violet was washed under running water and dried overnight. Digital images of the clones were obtained using a camera on the NIKON microscope (Tokyo, Japan).

### Crystal violet stains

Glioblastoma cell lines were seeded at 1 × 10^5^ cells per well in a 6-well plate. The cells were treated with varying concentrations of erlotinib and luteolin (10–20 µM) alone for 72 h. Additionally, in a different set of experiments, GBM cells were treated with a combination of erlotinib (0.125–0.5 µM) in the presence of luteolin (10 µM) for 72 h. At the end of the 72 h, the cells were washed with PBS and fixed with 4% paraformaldehyde for 30 min. These glioma cells were stained and incubated with crystal violet for 5 min. Cell images were captured using the NIKON H600L Eclipse microscope (Tokyo, Japan).

### Cell counts

U251 and U251 EGFR overexpressing cells were seeded at a density of 1 × 10^5^ cells per well. These cells were treated with either erlotinib (0–40 µM) or luteolin (0–20 µM) alone. In another set of experiments, these cell lines were treated with increasing concentrations of erlotinib in the presence of luteolin (10 µM) for 48 h. The cells were trypsinized and centrifuged at 120 x g for 5 min. The cell pellets were resuspended in media and stained with Trypan blue (0.4%). The viability was counted using CytoSmart (CytoSmart Technologies, Hillsborough, NJ).

### Cell cytotoxicity

A lactate dehydrogenase assay was performed to determine cell cytotoxicity as previously described in Smith et al., 2011. The release of lactate dehydrogenase by cells is a sign of cell injury. U251 MG and U87 and the corresponding cells overexpressing ΔEGFR cells were grown at a density of 1 × 10^4^ cells per well and were cultured in a 96-well plate and grown overnight. The cells were then treated with erlotinib (20 µM) in the presence of luteolin (10 or 20 µM) for 72 h. This intracellular LDH release was measured at 490 nm using the Promega Glomax MultiDetection System (Madison, WI). This experiment was done in triplicates.

### Hoechst and PI staining

U251 glioblastoma cell lines were cultured at a density of 1 × 10^5^ cells per well. The method of Ma et al., 2021 was used. Briefly, the cells were treated with erlotinib (10 µM) alone and in the presence of luteolin (10 or 20 µM) for 48 h. After treatment, the cells were washed with cold PBS and stained with Hoechst 33342 (5 µg) and PI dye (1 µg). The plates were placed in the dark for 15 min. Cell images were captured using the fluorescent phase-contrast microscope NIKON H600L (Tokyo, Japan) at a magnification of ×10.

### Analysis and quantification of apoptotic cells

Annexin V and propidium iodide staining is a versatile technique that is widely used for determining cellular death through apoptosis. To detect cell death, Annexin V/PI double staining kit (Thermo Fisher, Waltham, MA) was used for analyses according to the manufacturer’s protocol. The glioma cells were grown to 70% confluency and treated with either luteolin or erlotinib and a combination of both compounds. These cells were incubated with both Annexin V and PI for 15 min at room temperature in the dark, according to the manufacturer’s instruction (Thermo Fisher, Waltham, MA), and then subjected to flow cytometry to measure the rate of apoptosis (Crowley et al., 2016). The data generated by flow cytometry were analyzed using the BD Accuri C6 Plus software (Liu et al., 2014).

### Western blot analysis

Western blot analysis was performed as previously described (Stump et al., 2017; Anson et al., 2018). Following treatment with varying concentrations of luteolin and erlotinib, cells were washed with cold PBS containing 0.2 mM of sodium orthovanadate. Cells were lysed with a complete Triton-X-100 lysis buffer. Cell extracts were then centrifuged at 16000 x g for 10 min at 4°C. Protein concentrations were determined using a BCA^TM^ Protein Assay Kit Thermo Fisher (Westminster, MD). After quantification, cell lysates were treated with sample buffer and boiled for 5 min at 100°C. Equal amounts of protein (20 µg) were then loaded and separated on 10% SDS-PAGE. Following electrophoresis, the gels were then electroblotted onto polyvinylidene fluoride (PVDF) membrane, blocked using 3% Blotto for 1 h, followed by a diluted solution of primary antibodies against phosphorylated Akt, mTOR, p70S6K, NF kappa B, EGFR, STAT3. The membranes were incubated and rocked overnight at 4°C. Membranes were then washed three times with PBST. Following the washing, secondary antibodies (anti-mouse HRP or anti-rabbit HRP) were added to the membranes and rocked for 1 h at room temperature. Membranes were washed with PBST. The blots were then visualized by the Azure Biosystem 300Q imaging system using the enhanced chemiluminescence (ECL) reagent from Fisher Scientific (Milwaukee, WI) per manufacturer protocols. Densitometric analyses were determined by AzureSpot software version 2.2.167.

### Statistical analysis

All analyses were done using the GraphPad prism software version 9 (La Jolla, Ca), analyzing two-variable data with a simple t-test, and using a one-way analysis of variance (ANOVA) followed by the Bonferroni post hoc test. A value of *p* < 0.05 was considered statistically significant.

## Results

### Effects of luteolin and erlotinib on glioma cell growth

**FIGURE 2:**
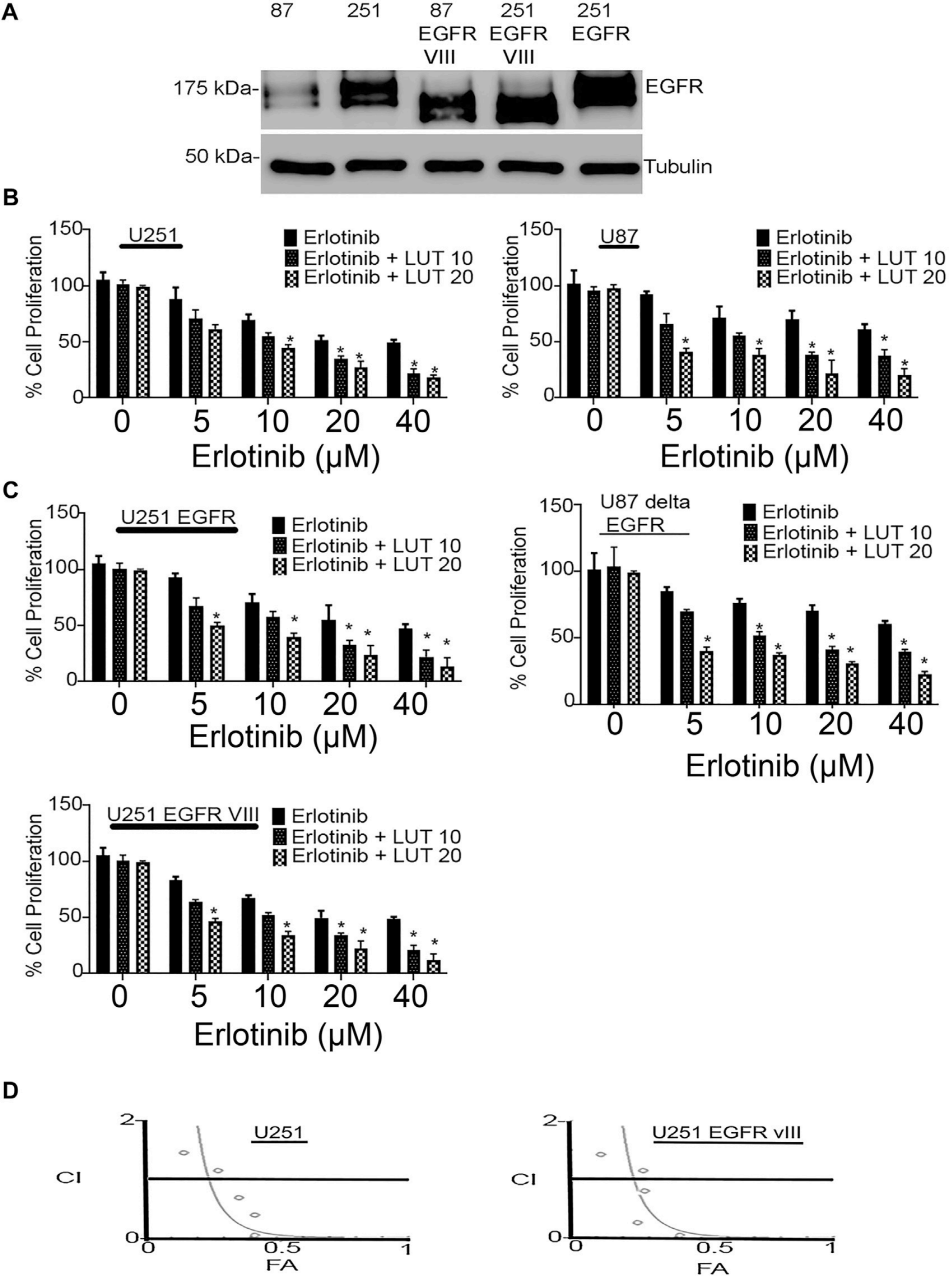
Antiproliferative effects of erlotinib and luteolin in glioblastoma cell lines. **(A)** Western blot analysis of the expression profile of EGFR, EGFRvIII in glioblastoma cells expressing the EGFR and the truncated EGFR. **(B)** Combined effects of erlotinib and luteolin treatment in U87 and U87 ΔEGFR induced a robust inhibitory effect on glioma cell proliferation as measured by MTT assay. The observed decrease in cellular growth was concentration-dependent in all the glioma cell lines tested. *p < 0.05 versus erlotinib alone. **(C)** Combined effects of erlotinib and luteolin treatment in U251 and U251EGFR, and U251 EGFR vIII induced a robust inhibitory effect on glioma cell proliferation as measured by MTT assay. The observed decrease in cellular growth was concentration-dependent in all the glioma cell lines tested. *p < 0.05 versus erlotinib alone. **(D)** Drug combination index showing synergistic effects of the combination of erlotinib and luteolin in glioblastoma cells. CI < 1, CI = 1, CI > 1 indicates synergistic, additive, and antagonistic effects, respectively.

**FIGURE 3:**
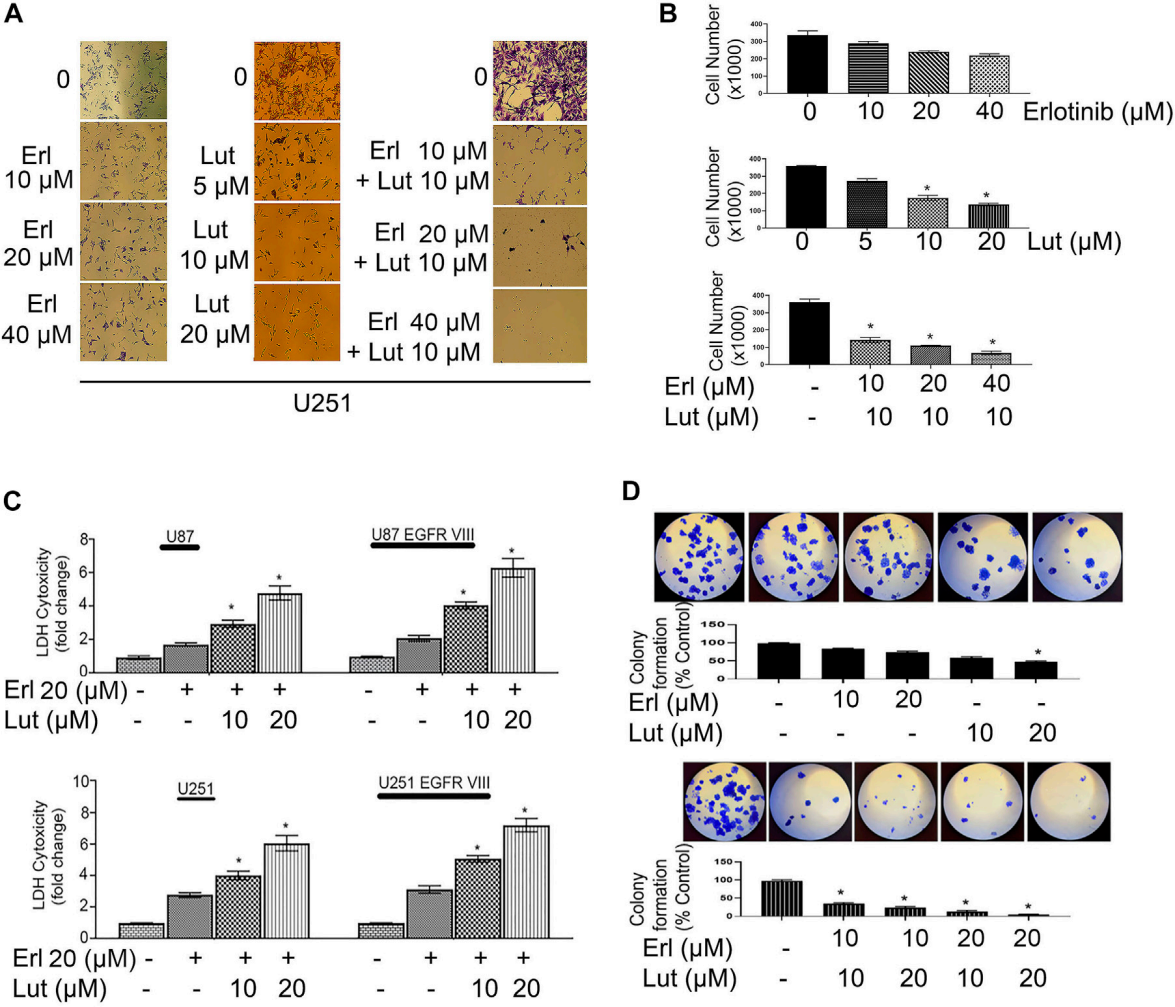
Effects of erlotinib and luteolin on cell morphology, cell counts, LDH assay, and colony formation assay. **(A)** The effects of erlotinib (0–40 µM) and luteolin (0–20 µM) on cell morphology. The combined effects of erlotinib (10–40 µM) and luteolin 10 µM on cell morphology. **(B)** The bar graph represents the number of viable cells following treatment with erlotinib or luteolin alone and the combination of both compounds. *p < 0.05 versus control. **(C)** The effects of erlotinib (20 µM) and luteolin (10 µM) on LDH release. The combined treatment showed a significant (*p < 0.05) increase in LDH release in glioblastoma cell lines. **(D)** The effects of erlotinib (10–20 µM) and luteolin (10–20 µM) alone on colony formation assay (upper panel). The combined treatment showed a significant decrease in the number of colonies formed in the U251 glioblastoma cell line (lower panel).

We first determined the EGFR expression profile in U251, U251 EGFR, U251 ΔEGFR, U87, and U87 ΔEGFR as shown in Figure 2A. Cell proliferation was determined using an MTT assay. Glioma cell lines U251, U251 EGFR, U251 ΔEGFR, U87, and U87 ΔEGFR were subjected to treatment with erlotinib alone (0–40 µM) and combined with luteolin (10 and 20 µM). As shown in Figures 2B,C, we observed that increasing the concentration of erlotinib minimally decreased cell proliferation. Our group and others have reported that the IC_50_ for erlotinib is 10 µM for U87; 30–35 µM for U251 and U87 EGFR vIII, (Wang et al., 2006; Schulte et al., 2013; Zhao et al., 2018; Alamdari-Palangi et al., 2020; Amini et al., 2021). The IC_50_ for luteolin was also found to be around 25–30 µM (Han et al., 2020). However, when combined with luteolin in increasing concentrations (10–20 µM), the overall cell proliferation percentage significantly (*p* < 0.05) decreased (Figures 2B,C). These observations remained true for glioma cell lines U87 and U87ΔEGFR, U251 EGFR, and 251 ΔEGFR. This result shows that not only does erlotinib minimally reduce cell proliferation, but the effects of combining luteolin and erlotinib also produce a more significant reduction (*p* < 0.05) in cell proliferation. We further subjected these drug combinations to test for synergism or additivity using the method of Chou-Talalay (Chou 2010). We observed that the combination of both agents provided a synergistic antiproliferative effect on the cancer cells (Figure 2D, and Supplementary Data S1). Additionally, we observed that treatment with erlotinib (0–40 µM) did not significantly decrease the cell viability and morphology (Figure 3A). Furthermore, our results showed that the combined treatment with both agents affects and alters cell morphology (Figure 3A) and decreases cell number represented as a percentage of cell viability (Figure 3B). Additionally, we investigated the effects of combining luteolin and erlotinib on the integrity of the cells and LDH release. Our results revealed that erlotinib at a concentration of 20 µM had a minimal increase in LDH release of about 2-fold compared with the control. Our data showed that the addition of luteolin (10 and 20 µM) significantly (*p* < 0.05) caused a 6-8-fold increase in cytotoxicity and the amount of LDH release (Figure 3C). We further investigated the ability of the individual compounds and their combination on cell reproduction potential as measured by the colony assay. Our data in Figure 3D clearly illustrates that combining the two agents significantly attenuated colony formation in glioblastoma cells.

### Effects of luteolin and erlotinib on cellular signaling

**FIGURE 4:**
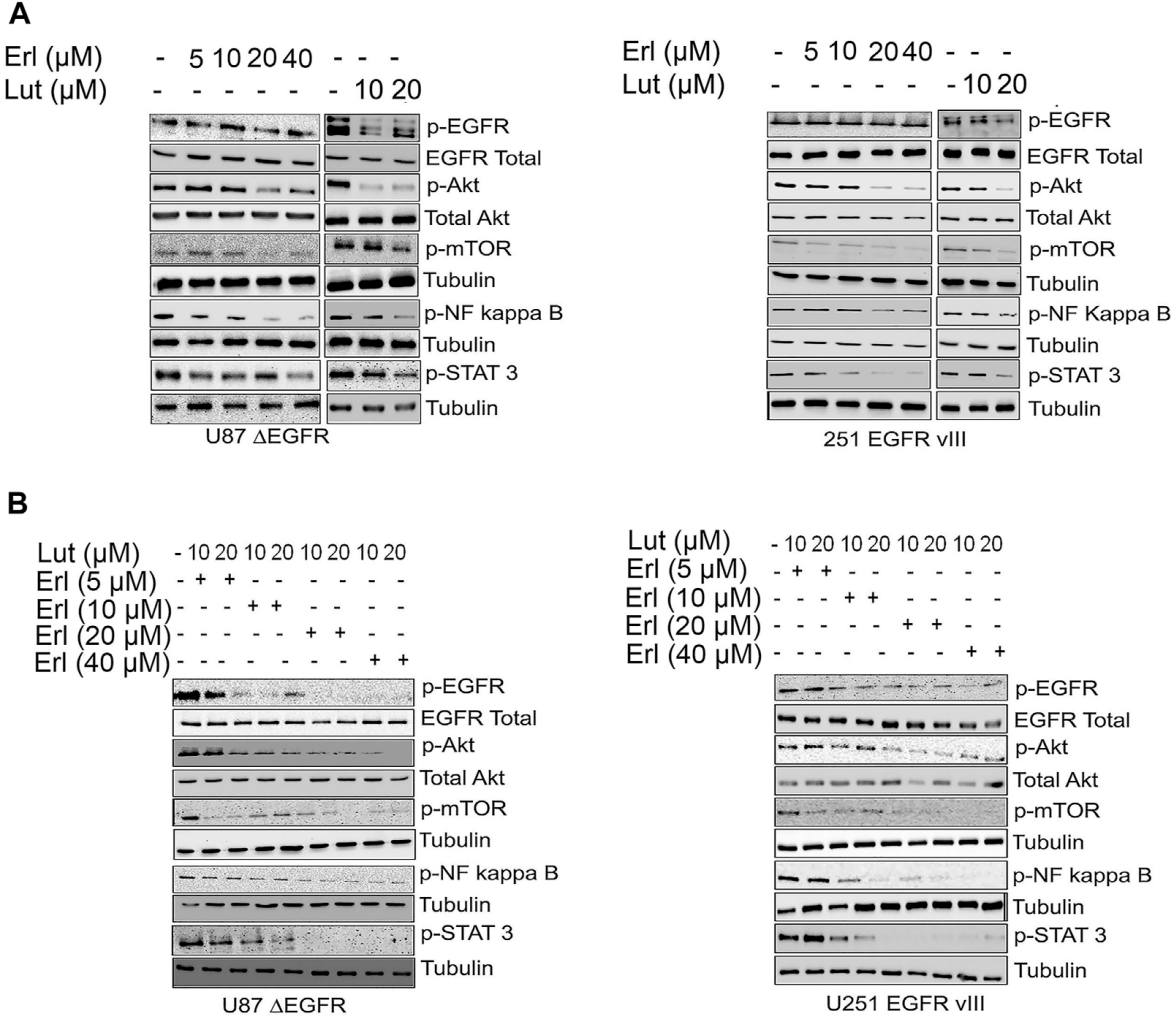
Effects of erlotinib and luteolin on growth mediated signaling proteins. **(A)**. The effects of increasing concentrations of erlotinib, luteolin (10–20 µM) alone and in combination on the expression profile of phospho EGFR, Akt, NF kappa B, and Stat3 in U87 ΔEGFR and U251 EGFR vIII. **(B)**. The combined effects of increasing concentrations of erlotinib and luteolin (10–20 µM) on the expression profile of phospho EGFR, Akt, NF kappa B, and Stat3 in U251 EGFR vIII.

This study utilizes the glioma cell lines that stably expressed the truncated EGFR (ΔEGFR). The expression of the ΔEGFR has been associated with chemoresistance and drug treatment failure (Padfield et al., 2015). Figures 4A,B show a western blot of glioma cell lines U87 ΔEGFR and U251ΔEGFR protein expression signaling after being treated with increasing concentrations of erlotinib, luteolin (10 and 20 µM), and the combination of the two compounds. We further investigated the effects of these drug combinations on EGFR downstream cell signaling proteins as they play an essential role in glioblastoma invasive growth. Increasing concentrations of erlotinib alone caused a minimal downregulation of p-Akt, p-mTOR, p-NF kappa B, and p-STAT3 cell signaling proteins. Similarly, at the concentrations tested, luteolin exhibited a minimal reduction in expression levels of p-Akt, p-mTOR, and p-STAT3 as compared with control. The combination of luteolin and erlotinib showed the most robust downregulation of survival cell signaling proteins. As we increased the concentrations of both agents, cell signaling proteins such as p-Akt, p-mTOR, p-NF kappa B, and p-STAT3 were significantly downregulated.

### Effects of luteolin and erlotinib on cellular apoptosis

**FIGURE 5:**
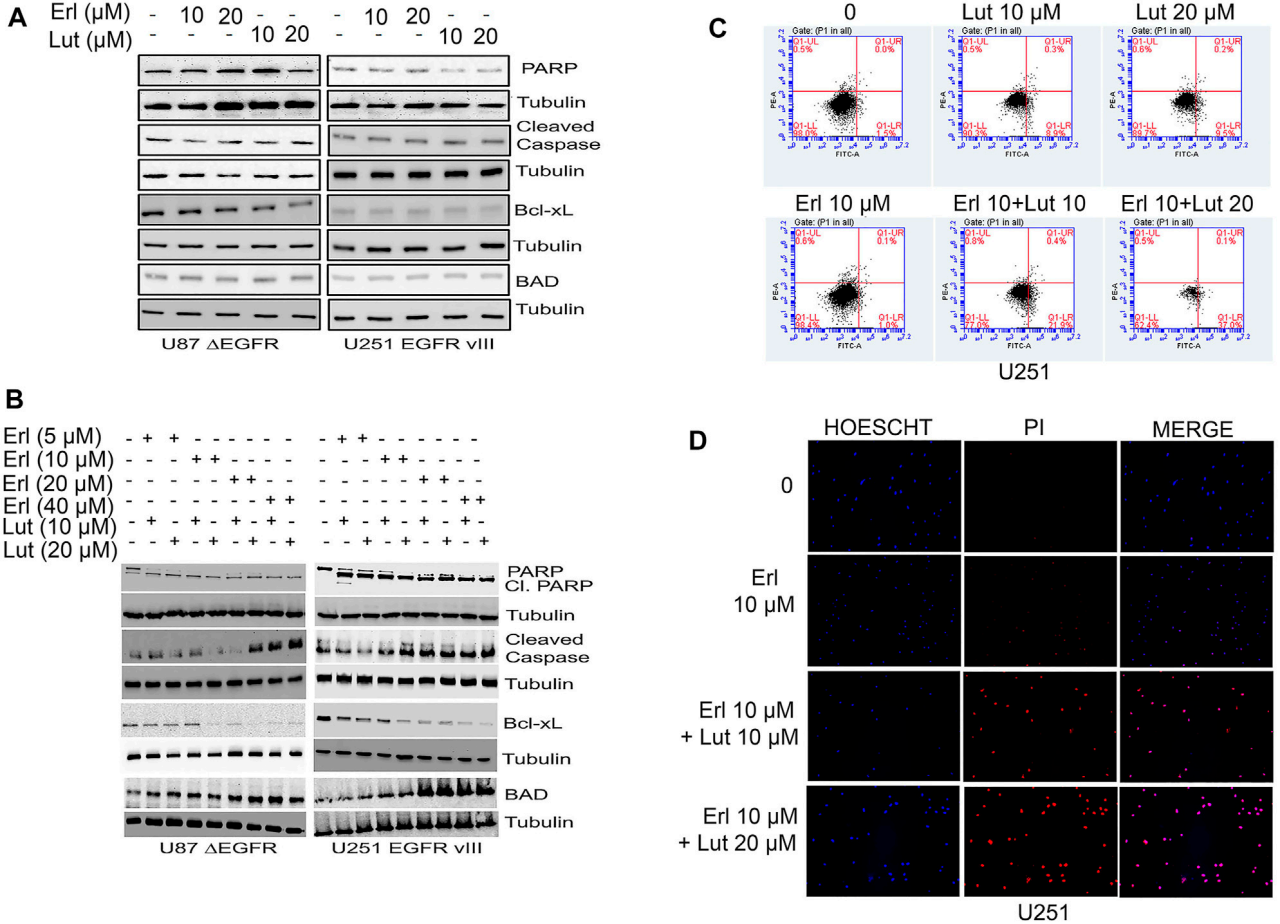
Effects of erlotinib and luteolin on apoptotic signaling proteins. **(A)** Effects of erlotinib and luteolin alone on key apoptotic signaling proteins such as PARP, Bcl-xL, and BAD on U87 ΔEGFR and U251 EGFR vIII. **(B)** Effects of combined treatment with erlotinib and luteolin on induction of key apoptotic signaling proteins such as PARP, Bcl-xL, and BAD on glioblastoma cells overexpressing the EGFR VIII (U87 ΔEGFR and U251 EGFR vIII). **(C)** Effects of erlotinib and luteolin on cellular apoptosis as measured by Annexin V and PI stain. The combined treatment with erlotinib and luteolin induced significant (*p* < 0.05) early apoptotic events. **(D)** Effects of erlotinib and luteolin on cellular staining with both PI and HOESCHT 33342. The combined effects of erlotinib and luteolin compromised the cells as evident by an increased PI stain.

In this set of studies, we investigated the effects of individual compounds and the combination of these small molecules on apoptotic cell signaling proteins such as PARP, cleaved caspase, and Bcl-xL. This study explored whether the combination of luteolin and erlotinib would increase apoptosis in glioma cells. Increasing concentrations of both erlotinib and luteolin were used and compared to control untreated cells. For glioma cell lines U87 ΔEGFR, and 251 EGFR VIII, we observed that erlotinib nor luteolin alone at the concentrations tested did not induce significant apoptosis as measured by the expression of cleaved PARP, cleaved caspase, Bcl-xL using Western blot analysis (Figure 5A). However, when these compounds were combined, we observed an increased expression of cleaved PARP, cleaved caspase, downregulation of Bcl-xL, and upregulation of BAD (Figure 5B).

Additionally, we tested the effects of erlotinib, and luteolin alone and in combination on cell apoptosis using the FITC-PI stains. We observed that treatment with erlotinib (10 µM) alone induced 1% early apoptotic cell death, while luteolin (10 µM), and luteolin (20 µM) induced a 9% and 9.5%, respectively. Furthermore, the combination of both erlotinib (10 µM) and luteolin (10 µM), erlotinib (10 µM), and luteolin (20 µM) produced robust cell death of 21% and 37% respectively, compared with control (Figure 5C). We also tested the effects of erlotinib and luteolin on cell integrity using Hoechst and PI stains in U251. We observed that treatment with erlotinib did not produce a significant PI stain, as this stain is taken up by cells with compromised cell integrity (Figure 5D). On the other hand, when these compounds were combined at the concentrations tested, we observed an increase in cells that take up the PI stain, suggesting that the combination of these compounds induced compromised cell integrity (Figure 5D).

## Discussion

Glioblastomas are among the most aggressive tumors. The high level of intra-tumoral heterogeneity within glioblastomas has contributed to the failure of EGFR-targeted therapies (Pan and Magge, 2020). As a consequence, cells are resistant to apoptosis and grow unchecked. We hypothesized that combining agents that target different signaling pathways would produce a synergistic effect on glioblastoma cells. In the current study, we investigated the combined effects of luteolin, and erlotinib to see if the combination would provide greater synergistic biological effects than either agent alone in glioblastoma cell lines. We found that the erlotinib and luteolin combination exhibited a significant decrease in cell proliferation, an increase in apoptosis, and a remarkable downregulation of growth-mediated signaling proteins.

As a lipid kinase, PI3K generates phosphatidylinositol-3,4,5-triphosphate. The PI3K signaling plays a crucial role in cellular functions such as survival, growth, and proliferation (Osaki et al., 2004; Vara et al., 2004). While Akt is involved in cellular proliferation by phosphorylating a variety of substrates (Liu et al., 2009). We hypothesized that cellular growth could be modulated by inhibiting the key regulators of these signaling pathways. Our results showed that even in cells expressing the truncated overexpression of EGFR (EGFR vIII), the erlotinib and luteolin combination decreased phospho-Akt, and phospho-mTOR, resulting in cell growth reduction. It has been demonstrated that Akt-mTOR pathways play a role in promoting growth, proliferation, and inhibiting apoptosis (Fruman and Rommel, 2014). Combining luteolin and erlotinib could lead to antiproliferative effects by downregulating these signaling molecules.

The PI3K/Akt pathway and activation of the EGFR, among others, can play a role in activating NF-kappa B (Fruman and Rommel, 2014; Colardo et al., 2021). NF-kappa B is an essential transcription factor linked to transduction pathways involved in cell proliferation and apoptosis (Wu and Kral, 2005; Fan et al., 2013). Based on our studies showed that p-NF kappa B was downregulated in cells overexpressing EGFR vIII and parental U87 cell line, resulting in decreased growth rate and increased cell death. It is possible that decreased expression of NF kappa B can lead to a delay in the progression of cells through the cell cycle (Yamamoto and Gaynor, 2001; Park and Hong, 2016), and subsequently result in a reduction in the decrease in cell growth and proliferation, as well as an increase in apoptotic cell death (Yamamoto and Gaynor, 2001).

Normally, pro-apoptotic and anti-apoptotic protein regulators maintain a balance during apoptosis (Pistritto et al., 2016). Reducing anti-apoptotic cell-signaling proteins increases cellular apoptosis (Zhang et al., 2020). It has been demonstrated that luteolin modifies the expression levels of proteins within the Bcl-2 family that contribute to apoptosis. The anti-apoptotic protein Bcl-xL promotes the survival of tumor cells (Loo et al., 2020). In both GBM cell lines (U87 ΔEGFR and U251 EGFR vIII), we found that Bcl-xL expression was downregulated, potentially resulting in the induction of apoptosis.

BAD, on the other hand, is a pro-apoptotic protein of the Bcl family. The western blot showed that the expression of BAD was elevated, which indicates that the combination of the compounds caused an apoptotic response. The higher the expression of BAD protein there is in the body, the more it can regulate apoptosis in the body, which results in cleaved PARP and caspase effects. When dephosphorylated, the BAD heterodimerizes with anti-apoptotic proteins Bcl-xL and Bcl-2 to promote apoptosis (Zhang et al., 2020). Bcl-xL is an anti-apoptotic protein that prevents cellular apoptosis. By decreasing this protein, cellular apoptosis can occur, which results in an increased apoptotic response (Loo et al., 2020). Our data suggest that the drug combination in our study induces apoptosis in glioma cell lines by decreasing Akt, downregulating Bcl-xL, and upregulating BAD.

Furthermore, PARPs/caspases also regulate apoptosis. As soon as caspases are activated, they begin cleaving several proteins that regulate cell survival (Chaitanya et al., 2010; Sastry et al., 2014). PARP is one of the substrates of caspase. Cleaved caspase three stimulates an apoptotic signal through enzymes on downstream targets such as PARP. PARP cleavage indicates apoptosis. Numerous diseases have been linked to caspase three cleavage of PARP (Sastry et al., 2014). In our study, luteolin combined with erlotinib exacerbated the effect of cleaved caspase three and PARP-induced apoptosis.

Prior studies have demonstrated that luteolin exhibits hormesis (Suraweera et al., 2020; Calabrese et al., 2021). Luteolin’s antioxidant properties potentially diminish the accumulation of reactive oxygen species that characterize glioblastomas and may contribute to its reported hormetic effects (Drake et al., 2003; Calabrese et al., 2012). Additionally, luteolin may activate or inhibit the vitagene network resulting in chemoprotection or apoptosis due to hormesis (Calabrese et al., 2007; Calabrese et al., 2010). When included in the diet or combined with other chemotherapeutic agents luteolin may lead to increased therapeutic efficacy (Suraweera et al., 2020; Calabrese et al., 2021).

Our studies showed that erlotinib, an EGFR inhibitor, when combined with luteolin, could induce apoptosis and decrease cell proliferation in glioblastoma cell lines expressing EGFR vIII. The effectiveness of erlotinib may be enhanced by the presence of luteolin, which reduces the growth and proliferation of cells, offering a new paradigm for the treatment of glioblastomas.

## Data Availability

The original contributions presented in the study are included in the article/Supplementary Material, further inquiries can be directed to the corresponding author.
