# Supplementary material for: Luteolin enhances erlotinib’s cell proliferation inhibitory and apoptotic effects in glioblastoma cell lines
Source: Front Pharmacol. 2022 Sep 19;13:952169. doi: 10.3389/fphar.2022.952169 (PMC9527275; doi:10.3389/fphar.2022.952169)
Supplement: Supplementary file 1 [file DataSheet1.PDF]

Supplemental data 1

A.

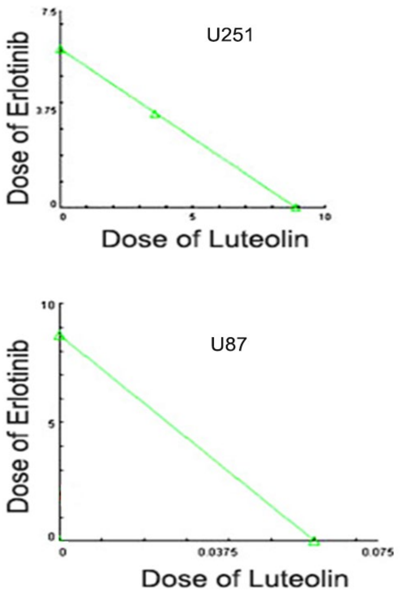

An isobologram analysis of the interaction between Erlotinib and luteolin using U251 and U87 glioblastoma cells. The diagonal line indicates additivity in cancer cell inhibition. Data from MTT assays for the combinations of the two drugs, were entered into Compusyn to produce normalized isobolograms that represent combinations of erlotinib and luteolin at the effective dose that induces inhibition of viable cell growth.

B.

CI values for actual experimental points:

Total Dose	Fa	CI Value
20.0	0.6	0.75289
20.0	0.54	0.62980
20.0	0.58	0.70889
40.0	0.42	0.88610
40.0	0.34	0.69159
40.0	0.32	0.64753

Total Dose	Fa	CI Value
80.0	0.25	1.00769
80.0	0.2	0.81743

DRI Data for Drug Combo: LE (L+Erl)

Fa	Dose L	Dose Erl	DRI L	DRI Erl
0.05	231.881	440.140	2.87946	5.46558
0.1	136.475	249.674	2.66496	4.87541
0.15	98.2929	175.765	2.54024	4.54241
0.2	76.7734	134.946	2.45021	4.30679
0.25	62.6005	108.484	2.37826	4.12143
0.3	52.3780	89.6509	2.31713	3.96604
0.35	44.5476	75.3946	2.26298	3.82999
0.4	38.2845	64.1158	2.21346	3.70692
0.45	33.1076	54.8886	2.16699	3.59262
0.5	28.7145	47.1367	2.12240	3.48405
0.55	24.9044	40.4795	2.07873	3.37876
0.6	21.5368	34.6539	2.03509	3.27458
0.65	18.5088	29.4698	1.99055	3.16936
0.7	15.7418	24.7835	1.94403	3.06064
0.75	13.1712	20.4810	1.89407	2.94524
0.8	10.7397	16.4648	1.83845	2.81848
0.85	8.38844	12.6411	1.77329	2.67228
0.9	6.04157	8.89905	1.69030	2.48976

Fa	Dose L	Dose Erl	DRI L	DRI Erl
0.95	3.55580	5.04808	1.56438	2.22092
0.97	2.43857	3.37239	1.48054	2.04749

DRI values calculated at experimental points

Fa	Dose L	Dose Erl	DRI L	DRI Erl
0.6	21.5368	34.6539	2.15368	3.46539
0.54	25.6272	41.7372	2.56272	4.17372
0.58	22.8379	36.8978	2.28379	3.68978
0.42	36.1032	60.2167	1.80516	3.01083
0.34	45.9683	77.9692	2.29842	3.89846
